# Age Influences Mid-term Survivorship in Unicompartmental Knee Arthroplasty: A 20-Year Single-Surgeon Cohort Analysis of 599 Patients

**DOI:** 10.7759/cureus.100331

**Published:** 2025-12-29

**Authors:** Sivashankaran Munuswamy, Kamparsh Thakur, Divya Prakash, Rohan Bassi, Varun Vig

**Affiliations:** 1 Trauma and Orthopaedics, Sandwell General Hospital, West Bromwich, GBR; 2 Orthopaedics, Armed Forces Medical College, Pune, IND; 3 Trauma & Orthopaedics, Sandwell & West Birmingham NHS Trust, Birmingham, GBR

**Keywords:** arthroplasty, isolated, lateral, medial, unicompartment

## Abstract

Introduction

Unicompartmental knee arthroplasty (UKA) is a well-established surgical option for isolated compartment osteoarthritis. Medial UKA is more common due to the higher prevalence of medial compartment disease. However, lateral UKA has shown promising results, although it is less frequently performed. This study evaluates mid-term outcomes in a large, single-surgeon cohort, comparing survivorship and revision rates across age, sex, and laterality.

Methods

Data were retrospectively collected from 599 patients undergoing primary UKA between 2004 and 2024 from the National Joint Registry (NJR). Patients were stratified by age, sex, and compartment (medial vs. lateral). Kaplan-Meier survival curves and Cox proportional hazards models were used to analyze predictors of revision. Revision, viability, and patient death were endpoints. Modern fixed-bearing prostheses were used in all cases.

Results

Among the 599 patients, 572 underwent medial UKA and 27 underwent lateral UKA. Mean follow-up was 5.6 years. The overall revision rate was 2.8%, all of which occurred in the medial group. No revisions were observed in lateral UKAs. Patients over 70 years experienced no revisions. Younger age was significantly associated with increased revision risk (hazard ratio (HR)=0.95, p=0.039).

Conclusion

With over 20 years of data, results demonstrate excellent survivorship, especially in patients over 70 years of age and in lateral UKAs, which showed no revisions. Age was a significant predictor of revision, whereas gender and surgical side were not. The study supports lateral UKA as a viable option when appropriately selected.

## Introduction

Osteoarthritis (OA) in the knee, based on its location, can be unicompartmental, bicompartmental, or tricompartmental. The most common pattern of arthritis is unicompartmental arthritis, involving 50% of the total population presenting with OA. Among the isolated unicompartmental arthritis, the medial tibio-femoral is most involved (27%), followed by patellofemoral arthritis (18%), and the isolated lateral compartment is the least commonly involved (5%) [1].

The surgical management options for isolated medial and lateral compartment OA include either a realignment osteotomy or unicompartmental knee arthroplasty (UKA) [2]. The osteotomy is generally indicated in symptomatic and young individuals [3], whereas UKA is indicated in symptomatic radiologically proven isolated medial or lateral compartment OA [4].

The outcomes of lateral UKA are comparable to those of its medial counterpart, as demonstrated in past studies. Both have shown excellent survivorship in the past. However, due to the lower incidence of lateral compartment OA, the volume of lateral UKA performed is very small, and thus comparisons between medial and lateral UKA are scarce [5]. At the same time, there have been significant advancements in the design of present-day prostheses. Most of the older prostheses used were mobile-bearing, which had complications of their own [6]. The modern prosthesis is a fixed-bearing design. Although the reported outcomes following a UKA are excellent, there is always concern that these patients may need a revision in the future. The most common reported cause for the revision is aseptic loosening [7].

The authors in this study present a large single-surgeon cohort of UKA over the past 20 years, with the primary objectives of examining survivorship and clinical outcomes. We further correlated age, gender, and laterality as variables that could affect survivorship and clinical outcome.

## Materials and methods

Study design and patients

This was a retrospective analysis. From the National Joint Registry (NJR), we obtained de-identified data on all primary unicompartmental (medial and lateral) knee arthroplasties performed by a single fellowship-trained arthroplasty surgeon between 2004 and 2024. Furthermore, their radiographs were scrutinized to identify and confirm the laterality. 

Inclusion and exclusion criteria

Inclusion criteria incorporated all adults who underwent primary unilateral unicompartmental (medial or lateral) knee replacement for any indication, irrespective of age, BMI, and presence of other significant medical comorbidities. Exclusion criteria included patients with bicompartmental or isolated patellofemoral joint replacement.

Data collection

The study was registered as an audit with the Trust Audit Module Database (via SafeGuard Webportal by Ulysses) by the ID number 3113. All cases were performed using a standardized medial or lateral parapatellar approach under spinal or general anesthesia. A cemented and fixed-bearing prosthesis was used in all cases. The sampling technique chosen was convenience sampling, including all consecutive cases. All cases were performed by the same surgeon either in a National Health Service (NHS) or a private hospital. After confirming the laterality, their current survival was noted in years from the date of surgery. The patients were divided into three age subgroups (A: <60 years, B: 60-70 years, and C: >70 years). The endpoint of the prosthesis was noted as either revision of the UKA (event=1) or survival of the prosthesis to date and death of the patients with the implant in situ (censored, event=0).

Hypothesis

The null hypothesis was that age, gender, and laterality would not affect survivorship and clinical outcomes. The alternate hypothesis was that age, gender, or laterality would jeopardize the survival and clinical outcome of the prosthesis.

Statistical analysis

JASP (University of Amsterdam, Amsterdam, Netherlands), open-source statistical software, was used to carry out the statistical analysis. Classical survival analysis methods were employed, with the approach integrating principles from machine learning. All standardized statistical tools were employed. Survival analysis was carried out using the non-parametric Kaplan-Meier estimator, which was further stratified by age groups. The log-rank test was used to compare survival among the age groups and test the null hypothesis. The Cox proportional hazards model was employed to estimate the hazard ratios for covariates (age, gender, and laterality) to identify predictors for revision of UKA. p-value <0.05 was taken to be statistically significant clinically.

## Results

A total of 599 patients were identified in our dataset between 2004 and 2025 who underwent UKA. Descriptive demographic and surgical-specific data of all patients included in the study are summarized in Table 1. There were a total of 572 medial UKAs and 27 lateral UKAs.

**Table 1:** Descriptive demographic and surgical-specific data summary

Gender	n	%
Male	334	55.8
Female	265	44.2
Age (years)	n	%
B (60-70)	247	41.2
A (<60)	216	36.1
C (>70)	136	22.7
Operated	n	%
Right	303	50.6
Left	296	49.4
Compartment	n	%
Medial	572	95.5
Lateral	27	4.5

Our three endpoints were defined as viable, implant revision, and patient death. Data showing this endpoint follow-up are summarized in Table 2. The mean follow-up time for viable implants was 5.6 years, and the mean time to revision was 3.7 years. There were no revisions for lateral unicompartmental knee replacements (UKRs), and no overall revisions were made to all the UKAs for those over 70 years of age. Data related to risk factors for revision and various indications for revision are summarized in Tables 3, 4, respectively. Indications for medial UKA revision included: unexplained pain, progressive arthritis, aseptic loosening of the tibial component, periprosthetic fracture, infection, and wear of the polyethylene component.

**Table 2:** Endpoint mean follow-up data

Endpoint	Cases	Mean follow-up (years)	Notes
Viable	541	5.6±4.4	90.3% of cohort
Death (patient died with implant in situ)	41	6.6±4.3	Deaths are censoring events for implant survival
Revised	17	3.7±2.6	2.8% crude revision rate

**Table 3:** Revision groups, counts, and risks within subgroups

Revision group	Subgroup	Number revised	Risk within subgroup
Gender	Female	9	3.4%
Gender	Male	8	2.4%
Age group	A	9	4.2%
Age group	B	8	3.2%
Age group	C	0	0%
Compartment	Medial	17	3.0%
Compartment	Lateral	0	0%
Side	Right	9	—
Side	Left	8	—

**Table 4:** Indicators for revision

Indication	Number	Average time to endpoint (years)
Infection	2	5.6
Unexplained pain	4	2.3
Aseptic loosening/instability	4	3.3
Malalignment	1	4.4
Progressive arthritis	2	2.1
Periprosthetic fracture	2	2
Polyethylene wear	1	7.8
Other	1	8.6

**Figure 1:**
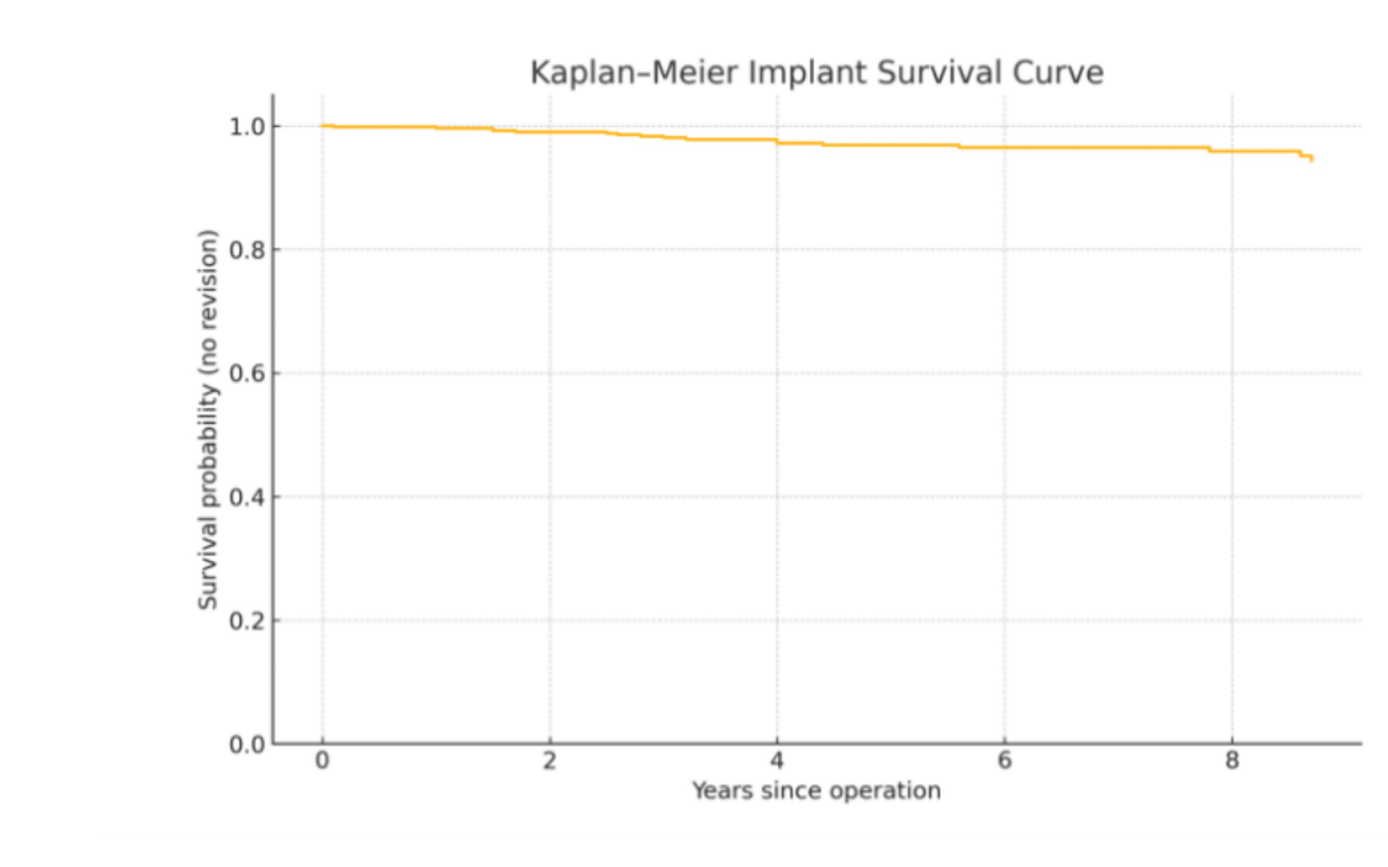
Overall Kaplan-Meier implant survival curve

A multivariate survival analysis examined age at surgery, sex, and laterality. The Kaplan-Meier curve below illustrates a high overall implant survival for UKAs, as shown in Figure 1, remaining over 95% over 10 years. The 10-year survival estimate for medial UKA was 555 out of 572 (94%) and 27 out of 27 (100%) for lateral UKA. Overall, only 17 (2.8%) medial UKAs were revised. Further, the Cox proportional hazard test determining age, sex, and laterality as potential cause for revision, with their significance, are described in Table 5. According to this, there was no statistically significant difference between genders for revision rates. However, there was a propensity for younger patients to undergo revision, which was statistically significant (although the absolute risk remained very low). Given there were only 27 lateral UKRs (limiting statistical power), no accurate statistical analysis can be made to compare the revision rates between medial and lateral UKRs.

**Table 5:** Cox proportional hazard test summary

Variable	Hazard ratio	95% CI lower	95% CI upper	p-value
Sex (F vs. M)	1.71	0.66	4.45	0.271
Age (per year)	0.95	0.9	1	0.039
Side (R vs. L)	0.92	0.35	2.4	0.857

## Discussion

This study presents one of the largest single-surgeon UKA series to date, offering valuable mid-term outcome data on 599 patients who underwent medial and lateral UKAs over a 20-year period. Our findings confirm that UKA, when performed with contemporary fixed-bearing implants, provides excellent mid-term survivorship, with an overall revision number of just 17 (2.8%), and no revisions were observed in the lateral UKA subgroup.

While medial UKA has historically dominated surgical volumes due to the higher prevalence of medial compartment OA (27% vs. 5% for lateral compartment disease) [1], recent studies suggest that lateral UKA may achieve comparable or superior outcomes when performed appropriately. Bai et al., in a large meta-analysis, found that lateral UKA was associated with equivalent or better functional outcomes and survivorship compared to medial UKA, particularly in younger, active populations [5]. This is corroborated by our results, in which zero revisions occurred in 27 lateral UKAs; however, the small sample size limits a definitive comparison.

Both Pandit et al. and Baker et al. reported 10-year survivorships exceeding 94% for lateral and medial UKAs in large registry analyses, which align closely with our Kaplan-Meier estimate of greater than 95% survival at 10 years [8,9]. Our medial UKA revision rate of 2.8% is substantially lower than the NJR average, which may be attributed to the standardized technique and implant consistency within a single-surgeon series.

Moreover, our data revealed no revisions in patients aged over 70 years, reinforcing the existing literature that links advanced age with a reduced implant failure risk due to lower activity levels [10]. Our Cox proportional hazards model confirmed this trend, showing a significant association between younger age and increased revision risk (hazard ratio (HR)=0.95, p=0.039).

Importantly, gender and surgical laterality (right vs. left) were not significant predictors of implant survival, consistent with findings from national registries, such as the AOANJRR (2024) [11]. Lateral UKA durability may stem from both careful patient selection and unique biomechanical factors. Unlike the medial side, the lateral compartment is subject to more natural load patterns and reduced varus stress, which can help minimize mechanical wear and loosening [12,13].

Another essential consideration is implant design. All patients in our cohort received fixed-bearing prostheses. Comparative data suggest that mobile-bearing UKAs, although designed to improve kinematics, have been associated with higher rates of complications, such as bearing dislocation and technical difficulties in the lateral compartment. Svard and Price reported increased rates of revision and bearing-related issues with early mobile-bearing designs [14]. In contrast, it has been observed to have improved outcomes and lower revision rates with robotic-assisted fixed-bearing UKAs [15]. Our zero-revision rate in lateral UKAs further supports the safety and efficacy of fixed-bearing designs in this setting.

Limitations

Despite the strengths of a large, single-surgeon cohort and consistent surgical technique, several limitations must be acknowledged. First, the low number of lateral UKAs (n=27) restricts the statistical power to perform subgroup analyses or draw definitive conclusions regarding comparative survivorship. This imbalance reflects real-world incidence but limits generalizability. Second, the retrospective design introduces inherent biases, including selection bias and information bias related to the reporting of endpoints. Third, while implant survival was the primary endpoint, functional and patient-reported outcomes were not assessed in this study, which are essential for a more holistic evaluation of UKA success. Fourth, despite the long study duration, long-term (15-20 year) data remain unavailable for most patients, particularly those with lateral UKA.

Future studies should include prospective, multicenter cohorts with standardized outcome reporting and larger lateral UKA samples to validate these early findings.

## Conclusions

This study demonstrates that UKA, particularly using modern fixed-bearing prostheses, offers excellent mid-term survivorship across both medial and lateral compartments. The absence of revision in the lateral UKA group, despite its small size, adds to the growing evidence supporting its use in appropriately selected patients. While medial UKA remains the more commonly performed procedure, lateral UKA is a reliable alternative that should not be overlooked, especially given the advancements in surgical technique and implant technology. Continued prospective data collection and reporting of functional outcomes are necessary to guide clinical decision-making.
